# Magnetic resonance imaging glenohumeral joint analysis before and after muscle transfer in children with obstetric brachial plexus palsy: a cross-sectional study of 10 cases

**DOI:** 10.11604/pamj.2024.49.34.43387

**Published:** 2024-10-11

**Authors:** Moez Trigui, Ahmed Racem Guidara, Imen Maaloul, Imen Zouche, Salma Ketata, Mohamed Habib Elleuch, Kheireddine Ben Mahfoudth, Hassib Keskes, Nizar Sahnoun

**Affiliations:** 1 Department of Orthopedic Surgery, Habib Bourguiba University Hospital, Sfax, Tunisia,; 2 Department of Radiology Habib, Bourguiba University Hospital, Sfax, Tunisia,; 3 Department of Anesthesiology and Intensive Care Unit, Habib Bourguiba University Hospital, Sfax, Tunisia,; 4 Department of Physical Medicine and Rehabilitation, Habib Bourguiba University Hospital, Sfax, Tunisia

**Keywords:** Obstetric brachial plexus palsy, magnetic resonance imaging, tendon transfers, glenohumeral deformity

## Abstract

This study aims to analyze the impact of muscle transfer on the glenohumeral joint in children with obstetric brachial plexus palsy (OBPP) using MRI by comparing preoperative and 5-year follow-up postoperative imaging findings to determine whether tendon transfers affect the alignment and configuration of the glenohumeral joint. Ten children with obstetric brachial plexus palsy (OBPP) participated in our prospective observational study, and we performed a tendon transfer technique. Every patient had an MRI of both shoulders done at preoperative and at the 5-year mark following the procedure. The glenoid form, glenoid version, humeral head hypoplasia (HHH), and percentage of the humeral head anterior (PHHA) were the parameters that were evaluated. The glenoscapular angle (GSA) was used to evaluate the glenoid version. Following tendon transfer surgery, MRI data show a regression of humeral head hypoplasia, and a statistically significant difference (p=0.0057) was observed between preoperative and postoperative results. Three individuals additionally recovered a normal glenoid shape. None of the remaining patients had a type 3 form; all had a type 2 form. Additionally, compared to the postoperative side, the damaged preoperative side's GSA was much more retroverted (p < 0.05). The mean PHHA for the afflicted shoulder was 25.8%, ranging from 0% to 40%, compared with 40.5% for the postoperative affected shoulder, indicating an improvement in humeral head subluxation (PHHA). As a result, the two results did not differ statistically. While tendon transfers led to only minor improvements in humeral head subluxation, they greatly improved glenoid retroversion and humeral head hypoplasia. It has been established that MRI is a useful diagnostic technique for glenohumeral anomalies due resulting from obstetric brachial plexus palsy.

## Introduction

With an incidence of 1 to 5 per 1000 births, neonatal brachial plexus palsy commonly causes osseous abnormalities of the glenohumeral joint and impaired muscular development of the rotator cuff muscles [1,2]. The paralysis of the abductors and external rotators, along with the relative hyperactivity of the adductors and internal rotators, generates an imbalance in the shoulder muscles that leads to the initial abnormalities of the glenohumeral joint. The medial rotators of the shoulder are comparatively “spared” in C5/C6 or upper trunk injuries, and internal rotation myostatic contractures of the subscapularis and pectoralis major are frequently present [2]. This early glenohumeral joint deformity is caused by this persistent internal rotational posture. Ultimately, subsequent subluxation or dislocation and even a permanent articular deformity of both the glenoid and humeral head are the consequences of these soft-tissue abnormalities surrounding the glenohumeral joint if treatment is not received. These abnormalities can be identified and quantified using MRI.

It is debatable how to address the shoulder disability in kids who still have brachial plexus palsy. Among the options are humeral osteotomy to realign the limb, tendon transfers to realign the glenohumeral joint, and open or arthroscopic reduction [3-5]. Theoretically, tendon transfers promote glenohumeral joint remodeling by redistributing forces around the joint [6,7]. Our study's objective was to evaluate the results of magnetic resonance imaging (MRI) following tendon transfers around the shoulder by comparing preoperative and 5-year postoperative imaging results. The objective was to determine whether tendon transfers affect the alignment and configuration of the glenohumeral joint.

## Methods

**Study design and setting:** this is a cross-sectional study with a before-and-after design, involving 10 cases. Conducted in the Orthopaedic Surgery Department and included children who presented to our hospital between January 2006 and September 2012 with Obstetric Brachial Plexus Palsy (OBPP) and operated for sub-scapular muscle release with a transfer of the teres major and the latissimus dorsi muscles.

**Study population:** patients who underwent muscle transfer surgery for OBPP were included. Exclusion criteria patients with bilateral OBPP, instances without a need for surgery, and individuals undergoing surgery other than tendon transfer finally we excluded patients with musculoskeletal affections.

Using a medical history and physical examination, we were able to confirm the presence of obstetric brachialplex palsy (OBPP) in newborns who came to our hospital between January 2006 and September 2012. The teres major and latissimus dorsi muscles were transferred, together with the sub-scapular muscle release, in all patients who underwent the identical surgical procedure.

**Sample size:** we estimate that a value of 50% of the value of humeral head anterior (PHHA) indicates that the head is in the exact center of the glenoid. The calculation was performed using a level (alpha) of 10% and a study power of 80%, which resulted in a minimum required sample size of 10 patients.

**Data collection:** data were collected from medical records, including patient demographics of participants, including age, gender distribution, and laterality of the affected side (left or right). surgical details, and MRI measurements before and after muscle transfer surgery. MRI scans were used to measure the alignment and configuration of the glenohumeral joint before and after 5 years of surgery.

**Operational definitions:** muscle transfer: the surgical procedure involving the relocation of a tendon from a functional muscle to improve movement. Alignment and configuration: alignment refers to the positions of the humeral head to the glenoid, and configuration refers to the shape of the glenoid and the humeral head form.

**Interventions:** all patients underwent MRI to assess the status of the glenohumeral joint before and after 5 years of undergoing shoulder surgical intervention. The final study sample consisted of 10 consecutive patients with OBPP. The extent of plexus palsy was categorized as upper palsy (C5 and C6 involvement), intermediate palsy (C5-C7 involvement), or global palsy (C5-T1 involvement). All MRI scans were obtained in the same 1.5 T MRI scanner (General Electric Medical Systems) with the use of the General Electric Advantage Workstation with version 4.4 reformatting software. Children younger than 6 years of age are imaged under general anaesthesia, according to our radiology and anaesthesia department guidelines for children of this age. Patients were placed in the supine position with both shoulders supported on the table and the arms relaxed in the neutral position. The imaging examination protocol for each shoulder included axial T1-weighted, axial T2-weighted, and axial fat-sat gradient-echo T2-weighted images (3.0 mm slices; 0.5 mm gap). The field of view was adjusted to the size of the child, and the matrix size was 256 x 256.

**Figure 1:**
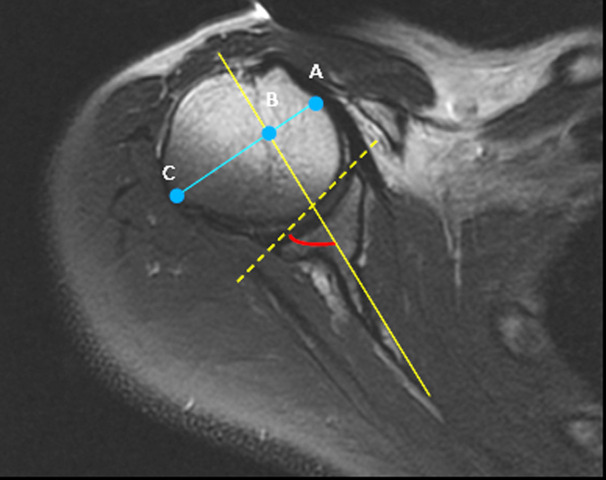
the method of measuring the glenoid scapular angle (GSA) and the percentage of the humeral head anterior

Parameters assessed focused on glenoid form, glenoid version, percentage of the humeral head anterior (PHHA), and humeral head hypoplasia (HHH). The glenoid form was classified according to the system proposed by Birch [8] (class 1: concave-flat, class 2: convex, and class 3: biconcave). The glenoid version was assessed using the method described by Friedman *et al*. [9] by measuring the glenoscapular angle (GSA) (Figure 1). One line was drawn from the medial margin of the scapula to the midpoint of the glenoid. A second line was drawn from the anterior to the posterior margin of the cartilaginous glenoid. GSA is the angle between the medial scapula line and the posterior glenoid line, subtracted by 90°. Both lines were drawn in the same image slice that represented the greatest glenoid girth, free from artefact. Positive values were interpreted as glenoid cavity anteversion, and negative values were interpreted as glenoid cavity retroversion. We estimated the severity of humeral head subluxation by measuring PHHA to the scapular line as described by Waters *et al*. [10]. We measured the distance between the margins of the humeral head by drawing a line perpendicular to the line of the scapula body (as in the Friedman method) at the midpoint of the humeral head. Thus, obtaining the ratio between the measurements (PHHA= AB/AC x 100) (Figure 1). A value of 50% indicates that the head is in the exact center of the glenoid, a value of less than 50% indicates varying degrees of posterior subluxation of the humeral head, and a value of 0% indicates that it is completely subluxated posteriorly.

For the surgical technique, patients were placed in lateral decubitus. We performed a broken axillary posterior approach, following the anterior border of the latissimus dorsi muscle and extending to the posterior surface of the arm. The teres major and latissimus muscles are disinserted from their common humeral insertion, and the subscapularis muscle is taken off its insertion into the anterior surface of the scapula [7]. This extraperiosteal detachment made it possible to obtain a gain of an external rotation up to an average amplitude of 45° (30 to 70°).

Then we reinsert the teres major and latissimus dorsi muscles on the rotator cuff (major tuberosity), more precisely on the infraspinatus. A postoperative immobilization by a thoracobrachial plaster, positioning the limb in external rotation of 45°, was put in place for six weeks.

**Statistical analyses:** statistical analyses were performed with SPSS for Windows (version 15.0) to compare parameters assessed between OBPP-affected limbs before and after shoulder surgical intervention. Descriptive statistics (mean) were calculated for the dependent variables; width (mm), inclination angle (°), 2-D version for each shoulder, and differences in retroversion between the affected shoulders in each child. For the quantitative variables, we checked the normality of the distribution by the Shapiro-Wilk test. Then we estimated the means with the standard deviations (SD) or medians with the semi-interquartile ranges (SIR) depending on the distribution.

A comparison between pre- and postoperative data focused on changes in alignment and configuration. It was done using the paired Student test for the comparison of means in the case of normal distribution and the Mann-Whitney test in the case of non-Gaussian distribution. Statistical significance of the correlations between variables was tested using either Spearman's rho in ranked variables or Pearson's rho in scaled variables. A P-value less than 0.05 was considered significant.

## Results

**Figure 2:**
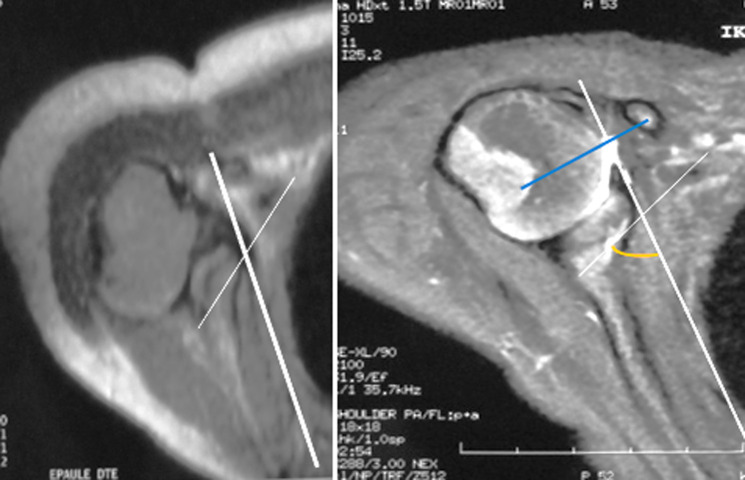
preoperative and 5 years postoperative MRI in a 3-year-old female with right residual brachial plexus palsy

**Figure 3:**
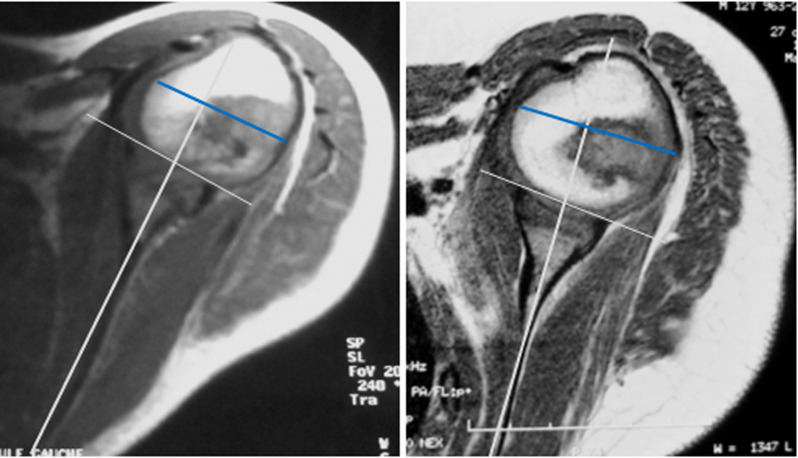
preoperative and 5 years postoperative MRI in a 5-year-old male with left brachial plexus palsy

In this study, ten children with unilateral OBPP were included with a mean age of 4 years (3 to 6 years), four girls and six boys. In four of the 10 children, the affected side was the left, and in six right. Table 1, Table 2, and Table 3 summarize the preoperative, 5-year follow-up postoperative MRI findings and correlations between results MRI-scan parameters which were calculated as shown in Figure 2, Figure 3. The preoperative report between the diameter of the affected humeral head and the diameter of the contralateral humeral head showed a value lower than 0.9 in 5 cases with a mean of 0.89. This implies that a humeral head hypoplasia was noted in 50% of cases. Compared with the post-operative report which was greater than 0.9 in all cases, we noted a statically significant difference between preoperative and postoperative results (p=0.0057<0.05), as can be seen in Table 3.

**Table 1:** preoperative magnetic resonance imaging results

Case number	HHH	Glenoid form	GSA	PHHA
1	0.86	3	-38°	0%
2	0.97	2	-18°	40%
3	0.92	2	-24°	28%
4	0.89	3	-30°	42%
5	0.85	3	-15°	37%
6	0.96	2	-19°	35%
7	0.87	3	-39°	16%
8	0.85	3	-56°	0%
9	0.9	2	-10°	360%
10	0.91	2	-28°	24%
Mean ± SD	0.89 ± 0.04		-27.70° ± 13.73	25.8% ± 15.68

HHH: humeral head hypoplasia; GSA: glenoscapular angle; PHHA: percentage of the humeral head anterior; SD: standard deviation

**Table 2:** postoperative magnetic resonance imaging results

Case number	HHH	Glenoid form	GSA	PHHA
1	0.96	2	-17°	38%
2	1	1	-10°	42%
3	0.98	2	-12°	40%
4	0.97	2	-13°	27%
5	0.99	1	-10°	46%
6	1	2	-12°	47%
7	0.96	2	-15°	44%
8	0.95	2	-27°	36%
9	0.95	1	-10°	45%
10	0.97	2	-14°	40%
Mean ± SD	0.97 ± 0.01		-14° ± 5.12	40.5% ± 5.93

HHH: humeral head hypoplasia; GSA: glenoscapular angle; PHHA: percentage of the humeral head anterior; SD: standard deviation

**Table 3:** comparison of preoperative and 5-year follow-up post-operative magnetic resonance imaging findings

Statistic	HHH		GSA		PHHA	
	Preop	Postop	Preop	Postop	Preop	Postop
Mean ± SD	0.89 ± 0.04	0.97 ± 0.01	-27.70° ± 13.73	-14° ± 5.12	25.8% ± 15.68	40.5% ± 5.93
P	0.0057<0.05		0.001<0.05		0.17>0.05	

HHH: humeral head hypoplasia; GSA: glenoscapular angle; PHHA: percentage of the humeral head anterior; preop: preoperative; postop: postoperative; SD: standard deviation; P<0.05 indicates statistical significance

In the affected shoulders on preoperative MRI, five patients had type 2-glenoid form and five had type 3 form. None of our patients had normal type 1 form. On postoperative MRI, we noted an enhancement of glenoid form: 3 patients had a normal type 1 form and 7 had a type 2 form.

The preoperative average glenoid version of the affected side was -27.7° ± 13.73 (range: -10° to -56°) versus postoperative -14°±5.12 (range: -10° to -27°). The mean GSA on the affected preoperative side was significantly more retroverted compared to the postoperative side (p< 0.05). The mean PHHA for the affected shoulder was 25.8% ranging from 0% to 40% compared with 40.5% for the postoperative affected shoulder. There was no statistical difference between the two results, which implies that the surgery did not modify significantly the degree of humeral head subluxation the posterior translation of the humeral head was significantly greater on the affected side (p=0.17).

## Discussion

In young patients, OBPP-related glenohumeral dysplasia is an infrequent cause of significant limb dysfunction. Ten OBPP cases that underwent tendon transfer shoulder surgery have been prospectively investigated. Treating the internal rotation contracture, expanding the range of abduction and external rotation, and averting secondary structural alterations in the glenohumeral articulation are the long-term objectives of shoulder surgery for brachial plexus palsy [10]. It is debatable how to address the shoulder disability in children who still have brachial plexus palsy. Progressive deformity will result from untreated muscular imbalance [11].

A typical trend in the treatment of late obstetric palsies with secondary glenohumeral dysplasia has been to undertake bone operations, such as humeral [12,13] and glenoid [14] osteotomies, as salvage procedures. This conclusion has been drawn because it is thought that soft tissue treatments might not be able to arrest or reverse glenohumeral joint bony alterations. On the other hand, tendon transfers potentially encourage glenohumeral joint remodeling by redistributing the forces around the joint [6]. Since this theory of muscle rebalancing leading to joint realignment is conjectural, more research utilizing cutting-edge imaging techniques is necessary.

With its perfect representation of the articular cartilage, humeral head location, and glenoid arrangement, MRI offers the most accurate imaging of the pediatric glenohumeral joint [15-19]. Therefore, for evaluating the alignment and configuration of the pediatric glenohumeral joint, this technique is recommended over computerized axial tomography scanning, ultrasound, and arthrography [16,17,19-22]. The literature has detailed a number of techniques for measuring and classifying glenoid dysplasia and the related findings. The accuracy and reproducibility of the procedures employed in this investigation are noteworthy; they have been previously detailed by Friedman *et al*. [9] and Waters *et al*. [17].

By utilizing MRI, Waters *et al*. [6,10] investigated the impact of tendon transfers and extra-articular soft tissue balance on the development of the glenohumeral joint. The clinical and radiological outcomes of twenty-five individuals with brachial plexus birth palsy who underwent tendon transfer with or without concurrent musculotendinous lengthening were evaluated. Every youngster showed signs of improvement in their clinical condition. Less noticeable radiographic alterations occurred. At follow-up, the mean glenoid version improved from 22 degrees of glenoid retroversion to 16.5 degrees, and part of this improvement might be attributed to typical growth-related changes in glenoid orientation [23]. The PHHA-evaluated humeral head subluxation improved from 30% to 37%. There was statistical significance in these gains. But even in young children, no significant glenoid remodeling was observed in the Scott and Kozin series [24]. Both prior to surgery and at the 3-year follow-up, they observed no change in glenoid retroversion [3]. Significant improvements in external rotation and abduction were observed in the clinical evaluation, however, humeral head subluxation (PHHA) remained unchanged. Numerous studies in the literature revealed conflicting findings on MRI evaluations, although they were all consistent in demonstrating notable clinical advances.

In the present study, MRI measurements showed a regression of humeral head hypoplasia after tendon transfer surgery and a statically significant difference between preoperative and post-operative results (p=0.0057<0.05) was noted. In addition, tendon transfers do appear to halt the progression of glenohumeral deformity. Indeed, three patients were able to recover a normal glenoid form. The rest of the patients had a type 2 form and none type 3. Moreover, in our study, GSA on the affected preoperative side was significantly more retroverted compared to the postoperative side (p< 0.05). Humeral head subluxation (PHHA) has been improved: the mean PHHA for the affected shoulder was 25.8% ranging from 0% to 40% compared with 40.5% for the postoperative affected shoulder. Therefore, there was no statistical difference between the two results; this implies that the surgery did not modify significantly the degree of humeral head subluxation.

## Conclusion

Bone and joint deformities of the gleno-humeral joint are frequent in OBPP children. Tendon transfers significantly improved in glenoid retroversion and humeral head hypoplasia but led to only modest improvements in humeral head subluxation.

### What is known about this topic

- Bone and joint deformities of the gleno-humeral joint are frequent in OBPP children;
- Magnetic resonance imaging (MRI) provides the most accurate image of the pediatric glenohumeral joint;
- Magnetic resonance imaging (MRI) precise depiction of the articular cartilage, humeral head position, and glenoid configuration.

### What this study adds

- Magnetic resonance imaging (MRI) is important in the evaluation of obstetric brachial plexus palsy sequelae in glenohumeral joints before and after treatment of by muscle transfer;
- Tendon transfers significantly improved in glenoid retroversion and humeral head hypoplasia;
- Tendon transfers led to only modest improvements in humeral head subluxation.
